# Gender Differences in Emergency Department Visits and Detox Referrals for Illicit and Nonmedical Use of Opioids

**DOI:** 10.5811/westjem.2016.2.29425

**Published:** 2016-04-28

**Authors:** Hyeon-Ju Ryoo, Esther K. Choo

**Affiliations:** * The Warren Alpert Medical School of Brown University, Providence, Rhode Island; † The Warren Alpert Medical School of Brown University, Department of Emergency Medicine, Providence, Rhode Island

## Abstract

**Introduction:**

Visits to the emergency department (ED) for use of illicit drugs and opioids have increased in the past decade. In the ED, little is known about how gender may play a role in drug-related visits and referrals to treatment. This study performs gender-based comparison analyses of drug-related ED visits nationwide.

**Methods:**

We performed a cross-sectional analysis with data collected from 2004 to 2011 by the Drug Abuse Warning Network (DAWN). All data were coded to capture major drug categories and opioids. We used logistic regression models to find associations between gender and odds of referral to treatment programs. A second set of models were controlled for patient “seeking detox,” or patient explicitly requesting for detox referral.

**Results:**

Of the 27.9 million ED visits related to drug use in the DAWN database, visits by men were 2.69 times more likely to involve illicit drugs than visits by women (95% CI [2.56, 2.80]). Men were more likely than women to be referred to detox programs for any illicit drugs (OR 1.12, 95% CI [1.02–1.22]), for each of the major illicit drugs (e.g., cocaine: OR 1.27, 95% CI [1.15–1.40]), and for prescription opioids (OR 1.30, 95% CI [1.17–1.43]). This significant association prevailed after controlling for “seeking detox.”

**Conclusion:**

Women are less likely to receive referrals to detox programs than men when presenting to the ED regardless of whether they are “seeking detox.” Future research may help determine the cause for this gender-based difference and its significance for healthcare costs and health outcomes.

## INTRODUCTION

Visits to the emergency department (ED) for use of illicit drug and misuse of opioid analgesic have increased disproportionately among certain sub-populations during the past decade.^1^,^2^ The number of ED visits for nonmedical use of opioid analgesics increased by 111% between 2004 and 2008^3^ in the setting of marked increase in opioid prescriptions from both primary care and ED.^4^ Interestingly, a clear gender-specific pattern has emerged from the epidemiologic data on opioid overdoses: the rate of drug overdose deaths increased 500% in women over the past decade, compared to a 360% increase in men.^3^ Drug use in the ED population is associated with high prevalence of comorbid conditions, injuries, high recidivism, morbidity, and mortality.^5^ Thus, the ED is an important point of contact for providing standardized screening, brief interventions and referrals to treatment (SBIRT) for drug use.^6^

In the face of modest outcomes from existing ED SBIRT studies, investigators have been turning to more targeted programs, reasoning that subgroups of patients might have different motivations for and barriers to changing drug use, responsiveness to specific intervention elements, and referral needs. Designing interventions based on gender is one promising avenue: gender is one of the most influential factors in determining trajectory of drug use disorders, accessing treatments, and achieving recovery.^7^–^10^ Outside the ED setting, a number of successful programs have been developed specifically targeting subgroups of men and women.^11^

Developing gender-specific interventions for the ED will require a better understanding of how men and women differ on various aspects of ED care. Frequently, studies include gender as a covariate but do not stratify samples or examine interactions between gender and other demographic or clinical variables; many studies explicitly studying gender are likely underpowered to detect differences between genders. Overall, there are few data on ED utilization by men and women with drug misuse and clinical outcomes of visits. As the opioid epidemic has demonstrated, following gender-related trends over time can shine a light on clinical problems that are manifesting in gender-specific or gender-sensitive ways, which in turn may represent gender-related vulnerabilities to adverse clinical consequences or special needs for prevention or treatment. Although there have been gender-based comparisons of utilization of substance-use treatment facilities and treatment outcomes,^12^,^13^ we know little about how treatment entry is facilitated and any gender disparity therein. The importance of emergency care research focused on gender disparities in patterns of referrals to substance-abuse treatments has been identified as a national priority.^14^

Our objective was to perform a gender-based comparative analysis of drug-related ED visits using a nationally representative database, Substance Abuse and Mental Health Administration (SAMHSA)’s Drug Abuse Warning Network (DAWN). Objectives were to examine, by gender the following: 1) rates of presentations and dispositions of patients presenting with use of major illicit drugs and nonmedical use of prescription opioids; 2) trends in drug-related visits over time; 3) patterns of referral to outpatient therapy upon discharge referred to as “detox referrals.”

## METHODS

DAWN is a nationally representative sample of ED visits to hospitals throughout the 50 states of the U.S.^15^ DAWN includes non-Federal, short-stay, general surgical and medical hospitals with a 24-hour ED.^15^ A retrospective chart review was performed by a trained DAWN reporter at each hospital. ED visits in which drug ingestion was the direct cause or contributing factor of the visit were identified.^15^ ED visits were codified based on a standardized algorithm – a “DAWN Decision Tree” – available in the *DAWN Methodology Report.*^16^ ED visits reportable to DAWN involve not only all forms of drug misuse and abuse but also adverse reactions, accidental ingestions, and visits where patients were seeking detox services.^15^ All drugs are classified based on DAWN Drug Reference Vocabulary, a drug coding system based on Multum *Lexicon*, © 2012.^15^ “Illicit” drugs are defined as cocaine, heroin, marijuana, synthetic cannabinoids, amphetamines, methamphetamine, Ecstasy (MDMA), gamma-hydroxybutyric acid (GHB), flunitrazepam (Rohypnol®), ketamine, lysergic acid diethylamide (LSD), phencyclidine (PCP), hallucinogens, or substances inhaled for psychotropic properties (e.g., sniffing model airplane glue). Nonmedical use of pharmaceuticals include misuse or abuse of prescription medications, over-the-counter medications, or dietary supplements that result from taking higher-than-prescribed dose, taking pharmaceuticals prescribed to another individuals, or malicious poisoning by another individual.^15^

This analysis used all available DAWN data, from 2004 to 2011, when data collection was terminated due to limited funding. Across the years, 2.6 million drug-related ED visits were identified: Applying post-stratified weights, these cases extrapolated to an estimate of 27.9 million drug-related ED visits out of an estimated 937 million total visits. DAWN captures limited data on individual visits, including age, gender, race, and disposition categories. It does not capture admission diagnoses, measures of illness severity, procedures performed, or length of stay.

Our analysis was restricted to adult patients (≥18 years of age). Individual drug types reported in the DAWN database were reviewed by the authors and further coded into categories of the following: 1) cocaine, 2) hallucinogens, 3) heroin, 4) marijuana; 5) methamphetamines; and 6) any illicit drug. We coded separately recreational drugs not clearly falling into one of these categories but included in DAWN’s definition of “illicit drugs” (e.g., cathinone and GHB). Drug combinations (such as cocaine/heroin) were placed in both categories. A single code for “any illicit drug use” was created that was positive if any of the previous drug categories were present and reflect “illicit drugs” defined by DAWN. We also created a variable for all prescription opioids that were included in the database for nonmedical use. All drug names under each category were reviewed by the authors, and any discrepancies between the two authors’ lists were resolved by using a toxicology database. Alcohol was a pre-defined variable in the DAWN database and only included underage drinking. Other variables of interest extracted for this study included gender, age, race, and clinical disposition, including 1) hospital admission or transfer; 2) intensive care unit (ICU) admission; 3) referral to outpatient detoxification; 4) admission for inpatient detoxification; and 5) psychiatric care.

As this study used only existing, publically available, de-identified data, it was exempt from institutional review board review.

### Data Analysis

We calculated proportions for demographic and drug use variables. The proportions for men and women were compared using unadjusted odds ratios.

Trends over time were presented graphically and examined using Stata 12.0 (StataCorp LP, College Station, TX). We developed logistic regression models to test for associations between gender and the binary outcome of referral to substance-use treatment programs among patients discharged from the ED. The first model adjusted for age, race, number of involved substances, and the time of day of the visit. These variables were determined a priori based on previous studies as characteristics available in the DAWN database that could potentially confound the effect of gender.^17^–^23^ The second model additionally included the chief complaint of “seeking detox.” This variable was selected after noting a difference between proportions of men and women “seeking detox” in the univariate analysis. Model variables were examined for evidence of collinearity with variance inflation factors (VIF), as high multicollinearity may lead to increased variance of model coefficients. Adjusted odds ratios (aOR) for which the 95% CI did not cross the null value of aOR=1.0 were considered statistically significant. For all analyses, we used “svy” commands in Stata to account for weights and clustering and to obtain accurate point estimates, standard errors, confidence intervals and tests of hypothesis. These have been described in previous studies.^24^ Model fit was evaluated using Hosmer-Lemeshow goodness of fit statistics.

## RESULTS

Of the 27.9 million ED visits related to drug use in the DAWN database 2004 to 2011, visits by men were 2.69 times more likely to involve illicit drugs than visits by women (95% CI [2.56, 2.80]). For every major category of illicit drugs, visits by men were more likely than visits by women to involve the illicit drug (Table 1).Visits by men were less likely to involve patients that were White (OR=0.75; 95% CI [0.70, 0.81]) and ages >55 years old (OR=0.75; 95% CI [0.72, 0.79]) as compared to women (Table 1).

Gender differences were observed in the ultimate disposition of ED patients presenting with drug use. ED visits by men were more likely than visits by women to result in hospital admissions with odds ratio of 1.19 (95% CI [1.14, 1.24]) (Table 1). Among discharged patients, men were 1.90 times more likely than women to receive detox referrals (95% CI [1.72, 2.09]) (Table 1). Among those seeking detox, 23.1% (95% CI [19.0, 27.2]) of women actually received referrals to detox compared to 24.5% (95% CI [20.7, 28.3]) of men. Conversely, among those discharged with detox referrals, 33.2% (95% CI [29.4, 37.1]) of women were “seeking detox” compared 41.4% (95% CI [38.2, 44.6]) of men. In other words, although a similar proportion of male and female patients “seeking detox” received detox referrals, “seeking detox” constituted a smaller proportion of women receiving referrals as compared to men.

**Figure 1:**
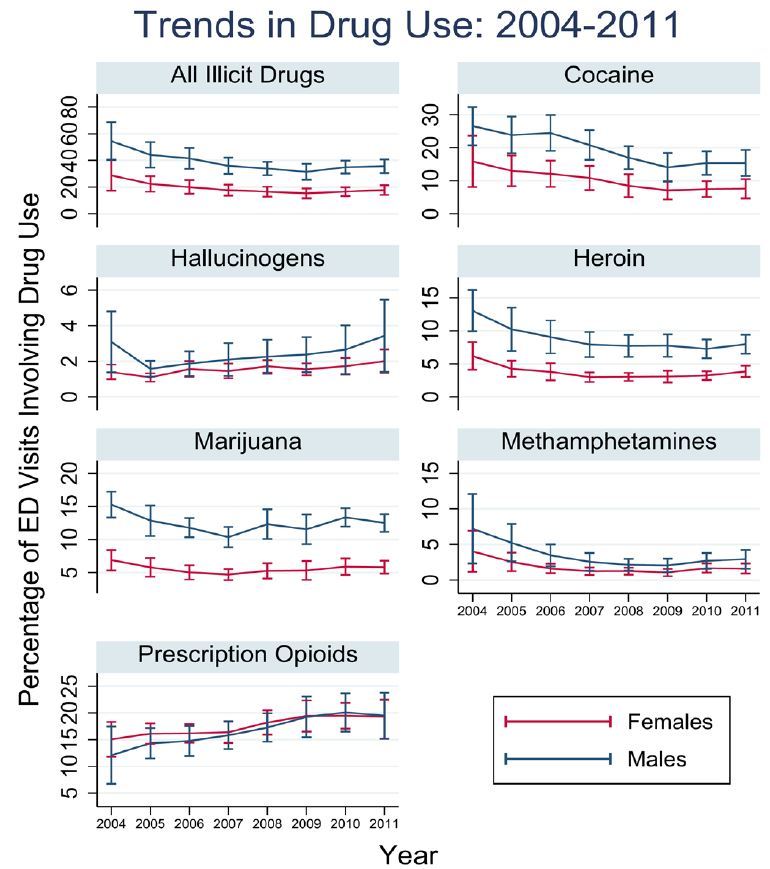
Trends in drug use: 2004–2011. *ED,* emergency department

For most years from 2004 to 2011, visits by male patients were significantly more likely to involve any illicit drug use, cocaine, heroin, and marijuana, as compared to women (Figure 1). Men and women had similar rates of visits involving hallucinogens, methamphetamines, and prescription opioids across all years studied (Figure 1). No significant changes in numbers of visits were found across the years for visits involving either male or female patients (Figure 1).

In logistic regression analysis, men were more likely than women to be referred to detox programs for any illicit drugs, each of the illicit drug categories, as well as prescription opioids (Table 2). This significant gender-based difference in detox referrals remained after adjusting for “seeking detox” for all categories of drugs, except for the combined category of “all illicit drugs” (Table 2). The VIFs were near unity, supporting lack of collinearity (Table 2).

## DISCUSSION

Approximately 23.9 million Americans aged 12 or older are current users of illicit drugs and the proportion of women using illicit drugs is growing faster than men.^25^ Women face many gender-specific barriers to treatment such as financial dependence, family responsibilities, and more frequent self-reports of shame and stigma.^7^ Given the potentially high impact of substance-abuse treatments, improving access to treatment services–particularly for women–is a public health priority.

Visits to the ED by women involving substance abuse present an opportunity to connect them to substance-use treatment and other mental health resources and address any existing barriers to accessing these resources. The DAWN database, however, demonstrates that nationwide, women are less likely than men to receive referrals to treatment for use of illicit drugs or misuse of prescription opioids. There are a number of potential causes for gender-based difference in referrals, including severity of drug use, comorbid conditions, patient motivation, and physician biases. The current literature, however, suggests that many of these factors should result in higher rate of treatment referrals for female than male patients. In particular, past studies have shown that women have greater severity of substance abuse upon presentation to treatment,^7^ and faster progression to dependence, also known as “telescoping.”^8^–^10^ The addiction literature also demonstrates that women are more likely to suffer from comorbid psychiatric conditions such as depression and anxiety and other health-related consequences than men; it is likely that women have greater needs for dual substance-use and mental healthcare facilities, which would address these interrelated problems simultaneously. Overall, these findings suggest that severity of drug use and comorbid conditions should have prompted more treatment referrals for women than men.

Our analysis indicates that patient motivation could have played a role in the fact that referrals were given more often to men than women. Although similar proportion of men and women “seeking detox” actually received detox referrals, more men than women with detox referrals were “seeking detox.” This descriptive finding suggests that motivation may play a larger role in obtaining referrals to treatments for men than for women. The regression model, however, demonstrates a greater rate of treatment referrals for men even after controlling for the chief complaint “seeking detox” for all drug categories. This finding suggests that lower rate of explicit request for treatment services by women may not entirely account for their lower rate of detox referrals for most categories of illicit drugs. On the other hand, men and women had similar rate of detox referral for “all illicit drugs” after controlling for “seeking detox.” One potential explanation is that “seeking detox” was an important reason for receiving detox referrals for patients using drugs that are part of “all illicit drugs” but not coded as major categories of illicit drugs, such as GHB, LSD, and PCP.

Prior studies have demonstrated physician bias in screening for substance use in the emergency setting.^10^,^26^ Similarly, past literature regarding alcohol treatment suggests that women are less likely than men to receive physician referrals to treatment centers.^7^,^27^ The higher prevalence of ED visits involving drug use among men as well as the higher admission rates found in DAWN database suggest that emergency physicians may be more aware of significant substance use among their male patients, and thus more vigilant about providing detox referrals for them. Physician- and healthcare-related barriers to detox referrals for women and interventions to overcome these barriers are important areas for future research.

Overall, the rate of referrals was very low: only 3.2% in women and 5.9% in men of those presenting with drug use problems. Although our analysis indicates that women are less likely than men to receive referrals, it is important to note that both genders have low rates of referrals and may therefore both be likely to face significant barriers to treatment. The low rate of referrals is consistent with past studies that have found that 27% of ED patients had unmet substance-abuse treatment needs^28^. The low rate of referrals reflects, in part, that the baseline population includes those who clearly did not present with drug abuse, such as those with accidental ingestions or adverse reactions. However, it may also demonstrate the limited availability of and referral to substance-use treatment programs, particularly for patients who are uninsured. Other barriers may include cost, lack of transportation, other family or work responsibilities, lack of information, functional differences arising from psychiatric illness, or domestic violence.^7^,^26^ It is also plausible that many of the patients presented with low amounts of drug abuse and were deemed not at risk enough to receive a referral. However, there is no “safe” or “low risk” amount of illicit drug use or nonmedical use of prescription drugs,and virtually all illicit drug users should receive some type of referral.

Over the past 40 years, there has been a dramatic improvement in the recognition of the specific needs of women with substance use disorders and the development of women-focused substance-abuse treatment programs^7^. Data from the National Treatment Improvement Evaluation Survey (NTIES), a study of publically-funded substance-use treatment units nationwide, demonstrated that women are more likely than men to receive individualized counseling and access-related services such as transportation and child care.^29^ Compared to men, women have comparable treatment retention, similar reductions in post-treatment substance abuse,^20^,^29^–^32^ and are less likely to experience relapse.^33^,^34^ Despite these advances, our analysis suggests that referrals in the ED may lag in recognition of substance use in women as suggested by our analysis and serve as a potential barrier to accessing substance-use treatments that have otherwise been shown to be efficacious for women.

## LIMITATIONS

Although the DAWN database is a useful resource for nationally representative drug-related ED visits, there are several limitations. The DAWN database relies on diagnosis of illicit drug use or misuse of opioids by ED physicians. Future studies with patients’ reports of illicit drug use and nonmedical use of opioids are needed to determine whether the gender disparity reflects the actual prevalence of drug use by patients in the ED or differences in the recognition of drug use by emergency physicians. The DAWN database was created as a surveillance system to monitor trends of drug use over time and thus poses several challenges in answering our clinical research question of gender differences in ED visits and detox referrals. Because the cases reportable to the DAWN database include only visits involving drug misuse, proportions are expressed as a percentage of DAWN cases rather than total ED visits. This does not allow estimation of the proportion of total visits with illicit drug use and may inaccurately reflect the differences between genders; specifically, it is vulnerable to underestimating visits by female patients, who are less likely to be identified as using illicit drugs^10^. The inherent limitations of data captured by the DAWN database also do not allow more in-depth inquiry regarding the severity of illness, patient level factors such as past drug abuse, socioeconomic status, or physical or mental health comorbidities, or physician-level reasons for failing to provide detox referrals. DAWN is not clear whether a referral to a dual diagnosis treatment center – a resource potentially more relevant to women than men, as discussed above – would have been captured as a referral to a substance-use treatment center; if dual diagnosis referrals were not categorized as substance-use treatment, this could lead to a systematic bias underestimating resources provided to women. It is unclear whether physicians did not recommend detox or whether the patient refused detox despite being offered a referral. A study with prospective data collection focused on capturing these details that were omitted in DAWN could better identify contributing factors to a detox referral or lack thereof. “Seeking detox” in the DAWN database is also a heterogeneous definition and includes a wide range of causes, such as experiencing withdrawal and request for medical clearance before entering jail or a detox program. More research is needed to investigate each cause for seeking detox and the impact of patient motivation in receiving referrals to detox. Specifically, future studies should stratify specific motivations for seeking detox (e.g., medical clearance for jail or for withdrawal symptoms) and their impact on detox referrals.

## CONCLUSION

The DAWN database suggests that nationwide, women are less likely to present to the ED and receive detox referrals for illicit drug use or nonmedical use of prescription opioids compared to men. Future research is needed to determine the cause for this disparity, including more in-depth investigation of patient- and physician-level factors leading to referral, its significance to clinical outcomes, and whether increasing referrals to detox programs for women in the ED may improve substance-abuse treatment use and other patient-centered patient outcomes.

## Figures and Tables

**Table 1:** Characteristics of emergency department visits related to drug use, by gender, from the Drug Abuse Warning Network (DAWN) database (N=27,865,483).

	Female proportion, %	Male proportion, %	Male:Female unadjusted OR (95% CI)
Age category			
18–29	25.3	30.0	1.15 (1.11, 1.18)
30–44	26.5	28.0	1.08 (1.04, 1.11)
45–54	16.7	18.2	1.11 (1.08, 1.15)
55 or older*	31.5	25.6	0.75 (0.72, 0.79)
Race			
White*	72.5	66.5	0.75 (0.70, 0.81)
Black/African-American*	17.4	20.9	1.25 (1.16, 1.34)
Other*	10.1	12.7	1.29 (1.17, 1.43)
Drug category			
Any illicit drug*	18.5	37.8	2.69 (2.56, 2.80)
Cocaine*	9.6	18.9	2.18 (2.06, 2.32)
Marijuana*	5.5	12.4	2.41 (2.31, 2.52)
Heroin*	3.6	8.6	2.59 (2.35, 2.64)
Methamphetamines*	1.7	3.3	1.95 (1.78, 2.14)
Hallucinogens*	1.6	2.5	1.54 (1.35, 1.76)
Prescription opioids	17.9	17.2	0.95 (0.90, 1.00)
Disposition†			
Hospital admission*	30.5	34.3	1.19 (1.14, 1.24)
ICU admission*	17.0	15.6	0.90 (0.85, 0.96)
Psychiatric admission*	4.1	6.5	1.62 (1.44, 1.83)
Discharged*	56.5	54.4	0.76 (0.72, 0.79)
Discharged with detox referral*	3.2	5.9	1.90 (1.72, 2.09)

*ICU,* intensive care unit

* Statistically significant difference between genders.

† Disposition categories do not add up to 100% because several disposition categories were omitted (e.g. transferred, deceased, left against medical advice). Admissions to the ICU and psychiatry are subset of the total hospital admissions. Omitted admissions include those to inpatient, surgery, and inpatient detox unit. Similarly, discharged with detox referral is a subset of detox. Discharged home and released to police/jail is not included in this table.

**Table 2:** Male:Female adjusted odds ratios (aORs) for referral to detox programs. Second column includes models adjusted for covariates used in first column in addition to an additional variable indicating if the patient presented to the emergency department with a complaint of “seeking detox”.

	Model 1 Male:Female aOR (95% CI)	Mean VIF	Model 2: “Seeking detox” Male:Female aOR (95%CI)	Mean VIF
Discharge with detox referral				
Any illicit drug	1.12 (1.02, 1.22)	1.05	1.06 (0.96, 1.17)	1.10
Cocaine	1.27 (1.15, 1.40)	1.06	1.13 (1.02, 1.26)	1.09
Hallucinogens	1.31 (1.19, 1.45)	1.02	1.14 (1.02, 1.27)	1.06
Heroin	1.23 (1.12, 1.35)	1.02	1.12 (1.01, 1.06)	1.07
Marijuana	1.30 (1.17, 1.44)	1.06	1.13 (1.01, 1.26)	1.09
Methamphetamines	1.31 (1.18, 1.45)	1.02	1.14 (1.02 1.27)	1.06
Prescription opioids	1.30 (1.17, 1.43)	1.04	1.13 (1.02, 1.26)	1.08

*VIF,* variance inflation factors
